# Targeting chronic cardiac remodeling with cardiac progenitor cells in a murine model of ischemia/reperfusion injury

**DOI:** 10.1371/journal.pone.0173657

**Published:** 2017-03-20

**Authors:** Janine C. Deddens, Dries A. Feyen, Peter-Paul Zwetsloot, Maike A. Brans, Sailay Siddiqi, Linda W. van Laake, Pieter A. Doevendans, Joost P. Sluijter

**Affiliations:** 1 Department of Cardiology, Experimental Cardiology laboratory, University Medical Center Utrecht, Utrecht, The Netherlands; 2 Netherlands Heart Institute (ICIN), Utrecht, The Netherlands; 3 Regenerative Medicine Center Utrecht, University Medical Center Utrecht, Utrecht, The Netherlands; University of Louisville, UNITED STATES

## Abstract

**Background:**

Translational failure for cardiovascular disease is a substantial problem involving both high research costs and an ongoing lack of novel treatment modalities. Despite the progress already made, cell therapy for chronic heart failure in the clinical setting is still hampered by poor translation. We used a murine model of chronic ischemia/reperfusion injury to examine the effect of minimally invasive application of cardiac progenitor cells (CPC) in cardiac remodeling and to improve clinical translation.

**Methods:**

28 days after the induction of I/R injury, mice were randomized to receive either CPC (0.5 million) or vehicle by echo-guided intra-myocardial injection. To determine retention, CPC were localized *in vivo* by bioluminescence imaging (BLI) two days after injection. Cardiac function was assessed by 3D echocardiography and speckle tracking analysis to quantify left ventricular geometry and regional myocardial deformation.

**Results:**

BLI demonstrated successful injection of CPC (18/23), which were mainly located along the needle track in the anterior/septal wall. Although CPC treatment did not result in overall restoration of cardiac function, a relative preservation of the left ventricular end-diastolic volume was observed at 4 weeks follow-up compared to vehicle control (+5.3 ± 2.1 μl vs. +10.8 ± 1.5 μl). This difference was reflected in an increased strain rate (+16%) in CPC treated mice.

**Conclusions:**

CPC transplantation can be adequately studied in chronic cardiac remodeling using this study set-up and by that provide a translatable murine model facilitating advances in research for new therapeutic approaches to ultimately improve therapy for chronic heart failure.

## Introduction

Translational failure of novel therapies for cardiovascular disease (CVD) is a substantial problem involving both high research costs and an ongoing lack of novel treatment modalities reaching the bedside [1]. Although the overall mortality for CVD declined in the past decade, no improvement in survival after the diagnosis of heart failure is observed [2]. With a 5-year mortality rate of 50%, the high need for new treatment modalities is accentuated.

Cell based therapies have been implied as a novel approach for cardiac salvage and myocardial regeneration. Ever since the first clinical application of stem cells for acute ischemic heart disease more than a decade ago [3, 4], various studies demonstrated tentatively promising results regarding quality of life and cardiac parameters [5–7]. Despite the progress already made in a short period of time, application of cell therapy for chronic heart failure in a clinical setting is still hampered by poor translation [8]. Recent meta-analysis data shows that cell therapy in small animal models results in an improvement in ejection fraction (EF) of 11%, which is lowered to 5% when applied in large animal models and even further decreased to 3% in clinical studies [5, 9, 10].

The problem of clinical translation of stem cell therapy is complex and reasons for potential translational failure are diverse, including applied cell source, injury model and timing of therapy [11, 12]. One particular reason for the difficulty to translate functional outcomes from small to large animal models (and eventually to the clinic) is that cell therapy in small animal models is predominantly investigated in an acute myocardial injury setting [13]. Only a limited number of studies [14–17] tested stem cell therapy in small animals during chronic cardiac remodeling before switching to pre-clinical large animal research. To allow for correct (pre-)clinical translation, it is of great importance to study the basic mechanisms behind cell therapy in small animal models during this chronic remodeling phase.

In this regard, small animal models are extremely valuable in pre-clinical therapeutic research as they are easily accessible, relatively cheap and easy to manipulate genetically. However, it remains difficult to apply local therapeutics in murine chronic heart failure models due to the lack of accessibility to the heart after invasive MI surgery and the difficulty to use injection catheters in the small vascular anatomy of mice. Therefore, in this current study we provide an integrated model of chronic cardiac remodeling in mice, where we make use of a minimally invasive echocardiography-based local delivery strategy [18, 19]. As a proof of concept, we evaluate the use of human cardiac progenitor cells (CPC), an exciting class resident heart progenitor [20, 21], in our developed murine chronic ischemia/reperfusion (I/R) model.

## Material and methods

(An expanded methods section is available in the S1 Appendix.)

### Meta-regression analysis

We used the dataset of our meta-analysis [9] on placebo-controlled CPC studies in MI in small animals and complemented the data with a variable for CPC therapy timing. The primary outcome left ventricular ejection fraction (LVEF) was used for this analysis. We defined the acute setting as CPC administration within 7 days after infarct, the sub-acute setting as between 7 and 28 days and a chronic MI model as CPC treatment after MI induction at 28 days or more. We used univariable meta-regression to test for a potential difference. Since this was a hypothesis-testing endeavor, we also included groups with less than 5 comparisons for our analyses of therapy timing.

### CPC isolation, expansion and transduction

Human fetal heart tissue was obtained by individual permission using standard written informed consent procedures and prior approval of the ethics committee of the University Medical Center Utrecht, the Netherlands. This procedure is in accordance with the principles outlined in the Declaration of Helsinki for the use of human tissue or subjects. CPC were isolated by using Sca-1^+^ conjugated magnetic beads as described previously [22]. Isolated CPC were able to differentiate into cardiomyocytes, endothelial cells and smooth muscle cells, confirming their stemness [23]. To facilitate identification *in vivo*, CPC were transduced with a lenti-viral construct, containing pLV-CMV-luc-GFP as described previously [24]. Cells were cultured in SP++ (M199, EGM2, FBS, P/S, NEAA) until 80% confluency and used for *in vivo* transplantation at passage 12-14.

### Animals

All experiments were carried out in accordance with the Guide for the Care and Use of Laboratory Animals, with prior approval by the Animal Ethical Experimentation Committee, Utrecht University, the Netherlands. As CPC of human origin were studied, immune compromised mice (NOD-SCID mice, Harlan Laboratories) were used to prevent graft reaction.

### Ischemia reperfusion model

Male NOD-SCID mice, aged 10-12 weeks, underwent left coronary artery (LAD) ligation as previously described [25, 26], followed by reperfusion after 60 minutes by releasing the ligature and removal of tubing. Reflow was confirmed by reversed discoloration of the heart.

### Cell transplantation model

**Fig 1:**
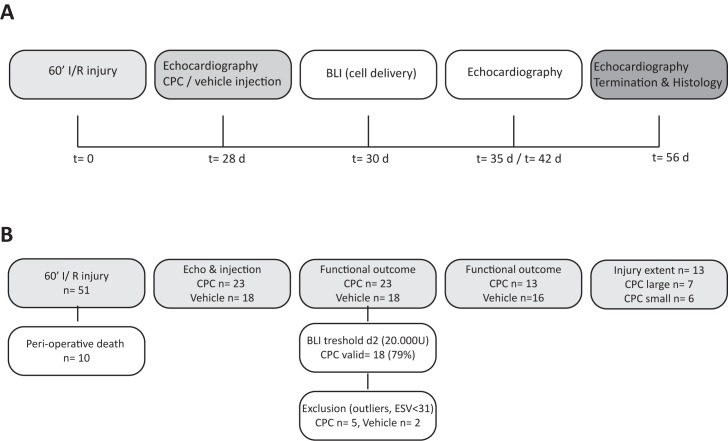
Study protocol. **A)** Timeline diagram of procedures and **(B)** flowchart of mouse experiment. Mice without significant cardiac damage (LVESV< 31), outliers or unsuccessful CPC injections (BLI signal after 2 days <20.000U) were excluded from primary analysis. For additional analysis, the CPC group was divided based on injury extent with the median LVEDV prior to cell treatment as cut-off point. ‘CPC small’ refers to animals with a LVEDV <median and ‘CPC large’ to animals with a LVEDV >median.

Twenty-eight days after induction of myocardial injury, animals were injected with either CPC, or vehicle (PBS) (Fig 1). To determine the extent of injury, mice underwent echocardiography (echo) followed by randomization in the different groups. Mice were positioned in an adjusted parasternal long axis view (PSLAX) for intramyocardial injection. CPC or vehicle were injected in the anterior wall via a transthoracic approach with echo guidance. A 27 Gy needle was used and 0.5 million cells were injected in two times 5 μl targeting the same ‘borderzone’ region.

### Bioluminescent imaging (BLI)

To determine the amount of engrafted CPC, emitted photons by CPC-luc were detected two days after injection by the photon imager from Biospace Laboratory and analyzed by Photovison software as previously described [24]. Injections were considered successful based on a threshold of BLI signal 2 days after injection (> 20.000 ph/s/cm^2^/sr), established with previously performed titrations [24]. After primary outcome analyses, animals with a low retention (< 20.000 ph/s/cm^2^/sr) were used for additional analyses.

### 3D motor echocardiography

Echo was performed at baseline, 28 days after I/R injury and at 7,14 and 28 days after treatment using a high resolution ultrasound system (Vevo 2100, VisualSonics) with a 18-38 MHz transducer (MS 400, VisualSonics). Echo acquisition and all analyses were performed by a blinded investigator. Left ventricular end-diastolic volume (LVEDV) and left ventricular end-systolic volume (LVESV) were used to calculate LVEF.

Pre-treatment echo measurements were analyzed and animals without significant cardiac damage, defined as an LVESV< 31 μl (= mean baseline value + 2x standard deviation) were excluded. Additionally, outliers were identified using the ROUT method (Q = 1%; [27]) and were excluded from further analyses. For additional analysis, the CPC group was divided based on injury extent with the median LVEDV prior to cell treatment as cut-off point.

Post measurement speckle tracking based analyses were performed to determine myocardial deformation parameters (VevoStrain, VisualSonics). Echo images acquired from the PSLAX were used to measure peak velocity (cm/s), strain (%) and strain rate (SR)(1/s) in the longitudinal and radial axis. For global measurements the average of all 6 myocardial segments (basal-anterior (BA), mid-anterior (MA), apical-anterior (AA), apical-posterior (AP), mid-posterior (MP) and basal-posterior (BP)) was taken. The infarct area was defined as the average of MA, AA and AP.

### Histological analysis

At day 28 after intramyocardial injection with either CPC or vehicle, mice were terminated by exsanguination under general anesthesia and their hearts were excised. The hearts were dehydrated and fixed in a 15% sucrose 0.4% PFA solution after which they were embedded in O.C.T. compound (Tissue Tek) and stored at -80°C. Serial transverse cryosections of 7 μm were cut, base to apex, for histological and immunohistological stainings. Imaging and analysis were performed by a blinded investigator.

### Statistical analysis

Statistical analyses were carried out using GraphPad Prism 6.0 software (GraphPad Software, La Jolla, USA). Data is presented as mean ± SEM and were compared using the two-tailed Student’s T-test. For analyses in time, the two-tailed paired Student’s T-test was performed. p< 0.05 was considered statically significant.

## Results

### Meta-regression

To systematically explore the effect of CPC in chronic cardiac remodeling, we performed meta-regression for the timing of therapy in our dataset of placebo-controlled small animal CPC studies for MI. The dataset contained 95 comparisons, of which 5 comparisons were in the sub-acute phase (1-4 weeks) and only 2 administered their therapy in a chronic MI setting (> 4 weeks after MI induction). Meta-regression showed a large spread in effect on LVEF for the timing of therapy (S1 Fig, p = 0.258). The low number of studies and large spread in effect endorsed our *in vivo* study.

### Cardiac engraftment and localization of CPC after echo guided intra-myocardial injection

A total of 51 mice were used in this study of which 10 mice died periprocedural. All other 41 animals were included in this study and 23 mice were injected with CPC (Fig 1).

**Fig 2:**
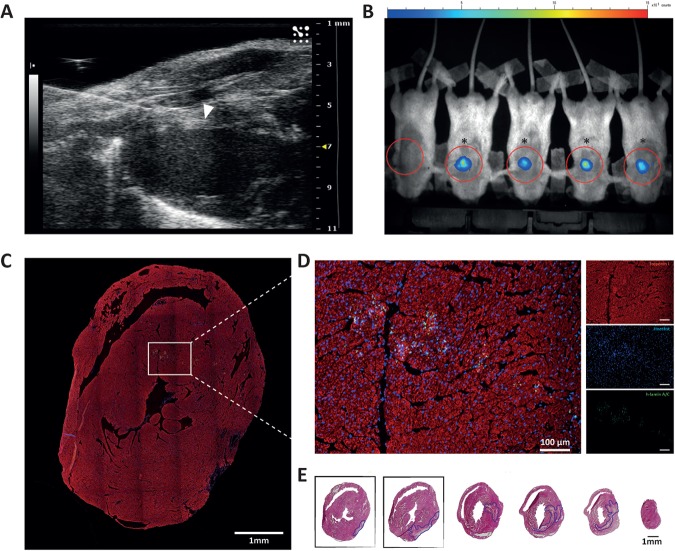
Echo-guided intramyocardial injection resulted in successful delivery of CPC. **A)** Representative B-mode image of intra-myocardial injection (29 Gy needle) at 4 weeks after the onset of I/R injury. The needle tip (marked by white arrowhead) is located in the anterior wall of the left ventricle. **B)** BLI images 2 days after intramyocardial injection with CPC demonstrated that 18/23 injections were successful (marked with *). **C-D**) CPC retained in the tissue up to 28 days after injection, as visualized by immunofluorescent staining for human lamin A/C (green), troponin I (red) and nuclei (blue). CPC were predominantly located along the needle track in the anterior and septal wall, at the level of the papillary muscles. (**E**) Squares mark H&E slides at the level of traced CPC. The left sided H&E slide corresponds to the CPC tracing slide in ‘C and D’.

To determine retention of transplanted CPC in the chronic remodeling heart, cells were localized *in vivo* by BLI two days after echo-guided injection (Fig 2A). Furthermore, cells were traced *ex vivo* with an anti-human lamin A/C antibody to confirm their presence and human origin. BLI showed a detectable signal in the targeted region in all CPC injected mice. Seventy-eight percent (18/23) of the injections were considered successful based on our previously determined threshold of BLI signal 2 days after injection (> 20,000 ph/s/cm2/sr [24], Fig 2B). CPC remained in the tissue up to 28 days after injection and were mainly located along the needle track in the anterior and septal wall (Fig 2C–2E). Transplanted CPC demonstrated an intact nuclear pattern and did not show clear tissue integration.

### I/R model in NOD-SCID mice

Pre-treatment echo measurements were analyzed and animals without significant cardiac damage, defined as an LVESV< 31 μl (= mean baseline value + 2x standard deviation) were excluded (n = 4). Additionally, 3 animals were defined as outliers using the ROUT method (Q = 1%; [27]) and were excluded. Representative echocardiographic images (three-dimensional reconstructions) of LVEDV and LVESV after I/R injury can be found in S2 Fig.

**Fig 3:**
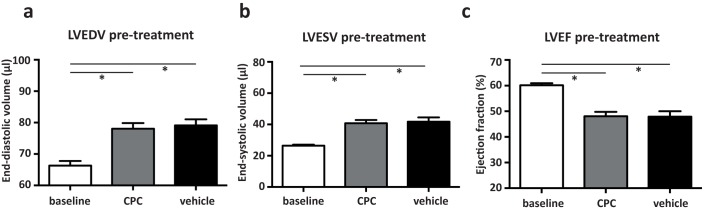
Functional measurements by echocardiography of day 28 (pre-treatment). Upon I/R injury the LVEDV (A) and LVESV (B) are increased, and the LVEF (C) is decreased with no differences between CPC treated or vehicle treated mice. CPC group: n = 19, Vehicle group; n = 16. * p< 0.001.

The geometry of the left ventricle was significantly altered upon MI, confirming successful induction of adverse cardiac remodeling. After 4 weeks, LVEDV (66.3 ± 1.5 μl to 78.5 ± 1.3μl) and LVESV (26.5 ± 0.7 μl to 41.2 ± 1.6 μl) were significantly increased (p< 0.001). Cardiac performance was affected by these changes, represented in a decrease in LVEF (60.1% ± 0.8 to 48.0% ± 1.3, p< 0.0001). No differences in cardiac geometry or function were observed between both experimental groups before treatment (LVEDV p = 0.78, LVESV p = 0.69, LVEF p = 0.95; Fig 3A–3C).

### Adverse cardiac remodeling is attenuated by CPC treatment

**Fig 4:**
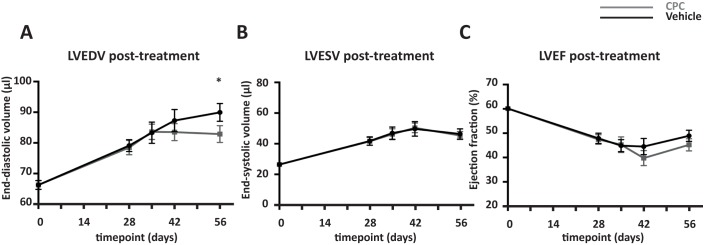
Functional outcome measurements by echocardiography at 1 month follow-up demonstrated preservation of the end-diastolic volume. **A**) LVEDV in the CPC group differed from the control group at experimental day 56 (post-treatment). No significant difference between the groups was observed for (**B**) LVESV and (**C**) LVEF. Black lines indicate vehicle treated mice (n = 16) and grey lines indicate mice treated with CPC (n = 13). * p< 0.05.

Treatment with CPC 28 days after I/R injury resulted in preservation of the LVEDV at 4 weeks follow-up (day 56 of experiment) compared to vehicle control (+5.3 ± 2.1 μl vs. +10.8 ± 1.5 μl, p = 0.036; Fig 4A). This difference was even more pronounced in mice with more left ventricular remodeling (+4.4 ± 2.6ul vs. +12.3 ± 2.5 μl, p = 0.045) compared to mice with a smaller injury (+6.3 ± 3.7 μl vs. +9.3 ± 1.7 μl, p = 0.43) (S3 Fig). To define this difference in extent of injury, we used the median LVEDV prior to cell treatment as cut-off point. Although not significant, the differential effect of CPC treatment was already observed 14 days after treatment (+4.7 ± 2.5 μl vs. +8.2 ± 2.2 μl, p = 0.3). Interestingly, this attenuation was not observed in the CPC group with low retention signals based on BLI two days after injection compared to vehicle control (+12.4 ± 5.0 μl vs. +10.8 ± 1.5 μl, p = 0.69; S3 Fig). In contrast to the observed differences in LVEDV, LVESV was uniformly increased in both CPC and vehicle treated mice (+6.4 ± 1.8 μl vs. +4.6 ± 1.7 μl, p = 0.48) and appeared to remain stable in time after therapy (Fig 4B). As a consequence, LVEF seemed slightly decreased in CPC treated mice (-3.9 ± 2.7%) compared to a minor increase (+1,26 ± 1.8%) in vehicle control (p = 0.16; Fig 4C).

### Matrix composition 28 days after CPC treatment

**Fig 5:**
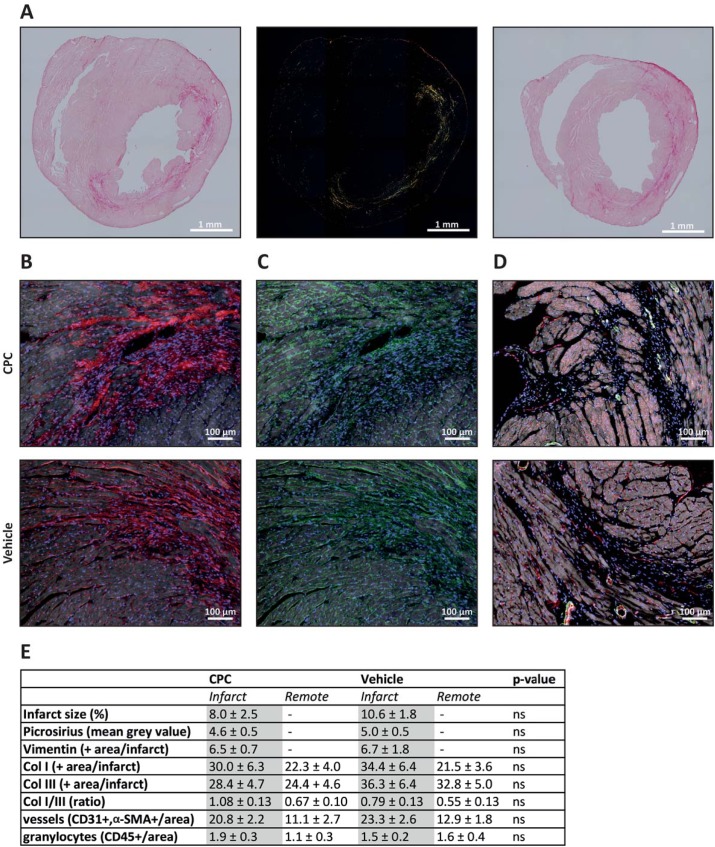
Histological analysis of matrix composition in the injured myocardium. Representative images of cryosections stained with picrosirius red used for quantification of the collagen density of the infarcted area (**A**). The left and middle panels show a brightfield and polarized light image of the CPC group, repectively. The right image represents the vehicle treated group. The collagen composition was assessed by immunofluorescent imaging of (**B**) collagen type I (red) and (**C**) collagen type III (green). (**D**) Immunofluorescent staining showed an increase of CD31^+^ (red) and a-SMA^+^ (green) vessels in the infarcted area compared to the remote area. No difference was observed between CPC or vehicle treated mice. Nuclei were stained with Hoechst (blue) and the myocardial structure is shown in grey. In **B-D** the upper panel represents the CPC group and the lower panel the vehicle group. (**E**) Table with quantification of the stainings.

Since CPC solely affected the LVEDV we sought to identify local effects of CPC on the infarcted tissue and extracellular matrix. The infarct size, defined as the percentage of non-viable left ventricle, was slightly lower in the CPC group (8.0 ± 2.5% vs. 10.6 ± 1.8%, p = ns). Accordingly, mice treated with CPC seemed to have a lower collagen density compared to vehicle-treated mice (4.6 ± 0.46 vs. 5.0 ± 0.54, mean grey value per mm^2^ infarct area, p = ns, Fig 5A and 5B). Further analysis of the composition of the extracellular matrix showed that although both groups had similar amounts of matrix producing cells (vimentin^+^ cells) in the infarcted area, the ratio of collagen type I/III appeared higher in mice treated with CPC (Fig 5A and 5D). All effects in matrix composition favored the CPC group, not reaching statistical significance (Fig 5E).

To determine if CPC increased the vascularization of the infarct area, like we demonstrated before in an acute injury model [21], a staining for CD31 and alpha smooth muscle actin (α-SMA) positive vessels was performed (Fig 5F). The staining demonstrated a higher vascular density in the infarcted area compared to the remote area (22.1 ± 1.7 vs. 12.1 ± 1.6, vessels/ mm^2^, p< 0.001). Treatment with CPC did not result in an increased vascularization (20.8 ± 2.2 vessels/mm^2^) in the infarct area compared to vehicle treatment (23.3± 2.6 vessels/mm^2^). In addition, no difference in CD45^+^ cells (granulocytes) was observed between the groups in the infarcted area.

### Speckle tracking analysis is sensitive for early changes in matrix composition

**Fig 6:**
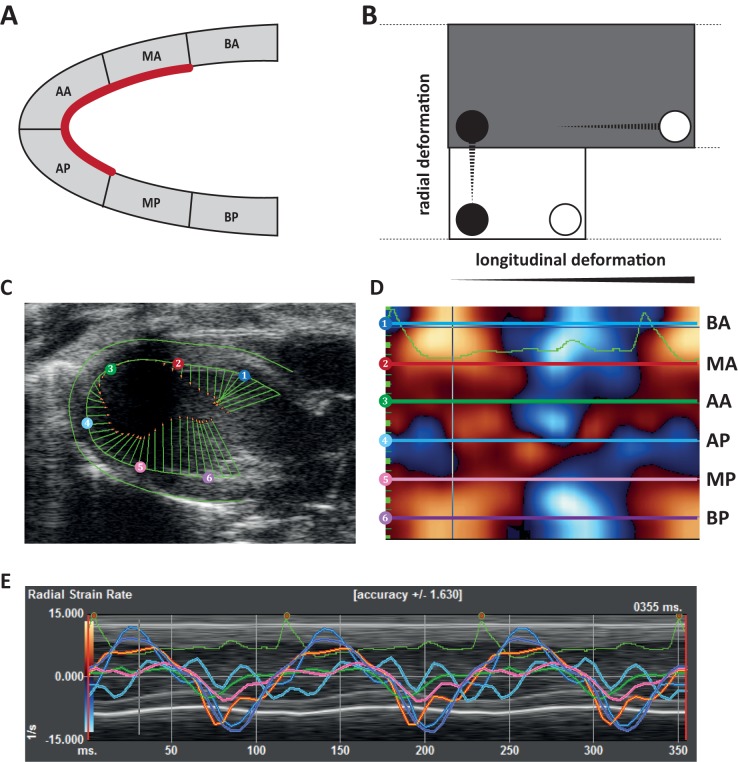
Myocardial deformation. (**A**) Schematic overview of 6 myocardial segments and deformation vectors; Basal-anterior (BA), mid-anterior (MA), apical-anterior (AA), apical-posterior (AP), mid-posterior (MP), basal-posterior (BP). Red line indicates infarct region. (**B**) Graphic representation of radial and longitudinal deformation. (**C**) Representative echocardiographic image of myocardial deformation (radial SR) after I/R injury, with concomitant vector lines, (**D**) for individual tracking points, and (**E**) combined with electrocardiogram and short axis view.

As our histological data demonstrated, no significant difference could be observed for the analyzed individual parameters between CPC or vehicle treated mice. In order to clarify the observed attenuation of the LVEDV, we used speckle tracking analysis for more detailed visualization of the regional wall motion dimensions of the myocardium (Fig 6A–6E). Upon I/R injury an expected decline in velocity, strain and SR was observed (Table 1). Global radial deformation changes were more pronounced than longitudinal deformation changes (S1 Table), with the latter being more affected in the infarcted region. Although no differences between the groups were found in conventional cardiac parameters (LVEDV and LVESV) prior to treatment, small differences were observed between the CPC group and vehicle group for radial velocity and SR, with a better performance for the vehicle group. Radial velocity was 0.7 ± 0.06 cm/s in the CPC group and 0.9 ± 0.06 cm/s in the vehicle group (p = 0.02). SR was 6.1 ± 0.4%/s in the CPC group vs. 8.0 ± 0.6%/s in the vehicle group (p = 0.01; Table 1).

**Table 1:** Regional myocardial deformation. Segments denoted as infarct area (MA, AA, AP) were used for quantification of velocity, strain and SR. The average value of the infarct area is given for the deformation parameters (velocity, strain and SR). p-values in the baseline column denote differences between baseline and 28 days I/R. In addition, p-values in the pre-treatment and post-treatment column show differences between the CPC and vehicle group.

Regional deformation	baseline		pre-treatment				post-treatment (delta)		
	overall		overall (n = 29)	CPC (n = 13)	Vehicle (n = 16)		CPC (n = 13)	Vehicle (n = 16)	
Radial									
velocity (cm/s)	1.1±0.08	**0.0002**	0.8±0.04	0.7±0.06	0.9±0.06	**0.0193**	0.1±0.04	-0.1±0.06	**0.0056**
strain (%)	35.4±3.3	**0.0007**	21.6±2.0	18.8±2.3	23.9±3.2	0.218	4.7±2.1	-1.2±3.1	0.1548
SR (1/s)	9.8±0.4	**0.0003**	7.2±0.4	6.1±0.4	8.0±0.6	**0.0131**	1.0±0.5	-0.7±0.6	**0.0409**
Longitudinal									
velocity (cm/s)	0.9±0.07	0.0829	0.7±0.05	0.7±0.1	0.7±0.05	0.6916	0.06±0.1	0.04±0.1	0.8709
strain (%)	-17.9±1.9	**0.0039**	-12.8±0.7	-12.1±0.9	-13.4±11	0.3713	-0.8±1.0	-0.08±1.1	0.6614
SR (1/s)	-7.2±0.6	**0.0046**	-5.3±0.3	-5.0±0.5	-5.5±0.5	0.4912	0.2±0.3	0.1±0.5	0.5984

To gain insight in the effect of treatment, the difference of post- and pre-treatment was calculated for all individual mice (Table 1). None of the longitudinal measurements were different between the two groups. However, analysis of myocardial deformation on the radial axis did show significant differences over time in favor of the CPC group. Radial velocity was increased in CPC treated mice (+0.2 ± 0.1 cm/s) while further decreased in vehicle treated mice (-0.1 ± 0.06 cm/s, p = 0.02). Likewise, strain and SR significantly improved for CPC treatment compared to vehicle. SR improved from 6.1 ± 0.4/s to 7.1 ± 0.6/s compared to a decrease (8.0 ± 0.6/s to 7.3 ± 0.6/s) for vehicle treatment.

## Discussion

Recently provided recommendations by the European Society of Cardiology, Working Group Cellular Biology of the Heart [20], aim to improve the therapeutic application of cell-based therapies for cardiac regeneration and repair by, among other recommendations, using more appropriate animal models that better resemble human chronic ischemic disease.

According to this, we tested CPC therapy in a clinically relevant mouse model of chronic cardiac remodeling. Minimally invasive intramyocardial injection of CPC was successfully applied 28 days after I/R injury and resulted in localized long-term engraftment. Although limited, transplantation of CPC resulted in attenuation of left ventricular dilatation and improved regional SR at 4 weeks follow-up with an effect size more closely resembling the results as obtained in large animal models and clinical studies. The challenge of clinical translation of cell therapy in small animal models is anticipated in this study by using an appropriate chronic I/R injury model with minimally invasive local therapy and clinically applicable functional outcome parameters.

We started off with a systematic assessment of the effect of CPC in sub-acute and chronic MI models compared to the acute setting. Our analyses show a large spread in effect size of CPC applied in the chronic setting and display a clear discrepancy in the number of small animal studies in chronic cardiac remodeling as compared to acute MI, which is worrisome due to the need for translation of this therapy to heart failure patients in particular. The observed translational failure in effect size might be attributed to animal size, but could also be caused by the models used, since 5 out of 11 placebo-controlled large animal CPC studies were done in chronic MI models, compared to only 2 out of 95 studies in small animal models [9].

Both Tang *et al*. and Tseliou *et al*. demonstrated an improvement of the LVEF upon treatment with CPC in a rat model of chronic cardiac injury with application of CPC 4 weeks after ischemic injury [14, 15]. Likewise, it was demonstrated that CPC treatment applied 7 days after permanent ligation of the LAD in mice resulted in improvement of LVEF and a lowering of the ventricular scar burden [17]. In contrast to these results, we found that the application of CPC in a murine chronic I/R model did not result in significant restoration of cardiac function. However, it is noteworthy that our results demonstrated an early preservation of the diastolic diameter of the left ventricle upon CPC injection.

The apparent discrepancy in treatment effects between performed studies, including ours, is most likely attributed to the timing of therapy, the used injury model, delivery method, source of CPC and cell retention. Our study was designed to mimic the human clinical situation of chronic ischemic heart failure with timing of therapy as a key prerequisite. Accordingly, we used a model of I/R injury to provide insight in the effect of human CPC in negative remodeling. To prevent immune reaction we used immune deficient (NOD-SCID) mice to accomplish that goal. Although a recent published report demonstrated that I/R injury in NOD-SCID mice does not result in sufficient cardiac damage [28], we show successful induction of cardiac remodeling upon I/R injury. Moreover, we administered local therapy 4 weeks after induction of MI to predominantly target chronic cardiac remodeling. In contrast, the previously described treatment at 7 days interferes with the extinguishing acute inflammatory response [29]. It is important to note that the therapeutic effect of cell therapy will be different in the distinct phases of cardiac injury and repair, e.g. tissue salvage in the acute phase and tissue remodeling in the chronic phase [12].

In line with the small beneficial results obtained in pre-clinical large animal research and clinical trials [30, 31], we demonstrated an attenuation of LVEDV increase upon treatment with CPC. In addition, there seems to be a difference in therapeutic benefit in mice with respect to pre-treatment extent of injury. These findings are in accordance with clinical meta-analysis demonstrating more effective treatment in patients with more severe cardiac dysfunction at the start of the therapy [32, 33].

With only two other placebo-controlled CPC studies in small animal models, the difference in beneficial effects remains difficult to explain and timing of therapy needs further exploration to better understand the role of the chosen injury model. In addition, it is known that CPC isolated with different isolation protocols display slightly dissimilar markers. However, this difference most likely did not influence the treatment effect since a high degree of similarity is found in their individual transcriptomes, although Cardiospheres displayed a higher secretory pattern of molecules involved in the development of cardiac muscle, vasculogenesis and angiogenesis [34].

Since we observed an effect of CPC treatment on eccentric cardiac remodeling, we assessed histological parameters to provide insight in the structural and cellular changes of the myocardial matrix. Although infarct size and collagen density appeared slightly smaller in the treated group, a direct link between histological parameters and the observed changes in cardiac volumes were absent. Since histological assessment is subject to a need for sample selection and thereby creates additional experimental variation or bias, the findings do not rule out that CPC interferes with matrix remodeling. Recently, it was demonstrated that CPC reduced fibroblast proliferation and attenuated pro-fibrotic signaling in a rat model of chronic MI [35]. This notion is supported by the observed long term improved left ventricular remodeling upon CPC treatment [14, 15, 36]. Several reports suggest that early changes in matrix remodeling are detected with a higher sensitivity with measurements of regional wall motion parameters by speckle tracking analysis [37–40]. Therefore, we assessed velocity, strain and SR of the infarcted region and indeed we have identified beneficial regional deformation changes upon CPC treatment, consistent with another study showing improved strain and SR upon myocardial treatment with induced pluripotent stem cells [41].

Implementation of regional wall motion measurements in small animal research might be valuable to identify more sensitive markers of cardiac function and cardiac improvement upon novel therapies. In parallel, these novel techniques merit further investigation to identify key modulators of myocardial remodeling translatable to pre-clinical large animal models and clinical cell therapy studies.

The resemblance of effect size with (pre-)clinical studies in literature underlines the translatability of our small animal injury model and shows that the apparent discrepancy in results can possibly be explained by the various approaches in the different models, of which timing of therapy and the type of injury model used are presumably the most important factors.

The model we present here can be exerted as a starting point to study the basic mechanisms behind cell therapy and to introduce more advanced methods in small animal research to examine therapies already tested in the acute post-MI phase. The tentative positive outcome of cell therapy in (pre-)clinical trials and the limited observed effects in our study force us to take the long view and to carefully address opportunities to optimize cell therapy to achieve clinical efficacy. Recently, tissue engineering approaches with the use of cell carriers improved initial cell retention and consequently resulted in a more beneficial treatment effect [42–44]. Furthermore, tailoring of cell differentiation protocols to direct cell faith is another promising approach to enhance therapeutic potential [45]. Therefore, exploration of different biomaterials and enhancing cell effectors would be of interest in future studies. To further maximize the success of preclinical research, a shift towards humanized small animal models should be made. By integrating aspects of the human immune system into mice models, a more comparable pathophysiology is created to study the multicellular interplay of cardiac remodeling upon I/R injury. Besides, co-morbidities and pharmacological agents should be incorporated in preclinical animal models [46–48]. The add-on effect of stem cell therapy will become clear when applied in combination with factors as hypercholesterolemia, diabetes, age, renal failure and gender [49].

Driven by the urge for clinical translation through proper animal models [50], the present study demonstrates a comprehensive small animal injury model to study chronic cardiac remodeling. We found that CPC transplantation can be adequately examined in this study set-up and by that provide a translatable small animal model facilitating advances in research for new local therapeutic approaches to treat chronic heart failure.

## Supporting information

S1 Appendix Supplementary methods. (DOCX)

S1 Fig Meta-regression of timing of CPC therapy in small animal models. Analysis of CPC studies in small animal models showed a large spread in effect on EF for timing of therapy (p = 0.26) with only 2 studies performed in the chronic MI setting.
(EPS)

S2 Fig Reconstructed 3D echocardiography. (A) Representative images of the contours of the LVEDV and (B) LVESV in a reconstructed 3D echocardiographic image. Bottom panels show examples of the contours drawn in the short axis images used to reconstruct the left ventricle.
(TIFF)

S3 Fig Subgroup analysis of functional outcome. (**A**) LVEDV is not preserved in mice with low retention at day 2 (unsuccessful injection). Black line indicates vehicle treated mice, grey line indicates mice with successful CPC injection and dotted grey line indicates mice with unsuccessful CPC injection. LVEDV = left ventricular end-diastolic volume. (**B**) The effect of treatment is higher in mice with a larger injury extent. Groups are divided by the median LVEDV as measured prior to treatment. * p< 0.05.
(EPS)

S1 Table Global myocardial deformation. All 6 myocardial segments were used for quantification of velocity, strain and SR. p-values in the baseline column denote differences between baseline and 28 days I/R. In addition, p-values in the pre-treatment and post-treatment column show differences between the CPC and vehicle group.
(DOCX)

## Abbreviations

CVD: cardiovascular disease
CPC: cardiac progenitor cell
EF: ejection fraction
I/R: ischemia reperfusion
LAD: left coronary artery
LVEF: left ventricular ejection fraction
LVEDV: left ventricular end diastolic volume
LVESV: left ventricular end systolic volume
ECG: electrocardiogram
SR: strain rate
PSLAX: parasternal long axis
SAX: short axis
BA: basal-anterior
MA: mid-anterior
AA: apical-anterior
AP: apical-posterior
MP: mid-posterior
BP: basal-posterior
α-SMA: alpha smooth muscle actin
