# Supplementary material for: Targeting chronic cardiac remodeling with cardiac progenitor cells in a murine model of ischemia/reperfusion injury
Source: PLoS One. 2017 Mar 20;12(3):e0173657. doi: 10.1371/journal.pone.0173657 (PMC5358772; doi:10.1371/journal.pone.0173657)
Supplement: S1 Appendix — (DOCX) [file pone.0173657.s001.docx]

**S1 Appendix**

**Supplementary methods**

**Animals**

All experiments were carried out in accordance with the Guide for the Care and Use of Laboratory Animals, with prior approval by the Animal Ethical Experimentation Committee, Utrecht University, the Netherlands. Mice were housed in filtertop cages with 5 mice per cage and cages were enriched with tissues. In addition, water and food was provided ad libitum. Regular day night cycles were maintained and mice were checked daily.

**Ischemia Reperfusion model**

CPC of human origin were studied and to prevent graft reaction, immune compromised mice (NOD-SCID mice, Harlan Laboratories) were used. Male NOD-SCID mice (Harlan Laboratories), aged 10-12 weeks, underwent left coronary artery (LAD) ligation as previously described (21), followed by reperfusion. In short, mice were anesthetized with Fentanyl 0.05 mg/kg, Midazolam 5 mg/kg and Domitor 0.5 mg/kg trough an intraperitoneal (ip) injection. Mice were intubated and ventilated with a mixture of oxygen and air (1:2) with a rate of 250 ventilations/min. Before any incision was made, de adequacy of the given analgesia and anesthetics was monitored by testing rear foot reflexes. During the procedure the respiratory pattern, rectal temperature and responsiveness to manipulations were monitored. The thorax was opened between the 3^th^ and 4^th^ rib and the LAD was ligated 2-3 mm below the left atrial appendage with a 8-0 Ethilon suture (Ethicon) around a soft tube (BD insyte-WA). After 60 minutes, reperfusion was initiated by releasing the ligature and removal of the tube. Reflow was confirmed by reversed discoloration of the heart. The chest was closed in layers and anesthesia was antagonized by a mixture of Antisedan 2.5 mg/kg and Anexate 0.5 mg/kg injected subcutaneously. The first 48 hours after the procedure, mice received analgesia (0.15mg/kg Temgesic) twice a day via a subcutaneous injection. During this period, a heating blanket was placed under the cage for appropriate environmental control and food and water was placed inside the cage to promote accessibility. In addition, mice were weighted and were checked daily for signs of severe illness to determine whether early humane endpoints were reached. Criteria included weight los of >15% in the first two days, weight los >20% during the entire experiment or strongly reduced mobility or activity.

**3D motor echocardiography**

Echocardiography (echo) was performed on baseline, 28 days after I/R injury and at 7,14 and 28 days after treatment using a high resolution ultrasound system (Vevo 2100, visual sonics) with a 18-38 MHz transducer (MS 400, visual sonics). Physiological parameters (heart rate, respiration and temperature) were monitored continuously and were maintained stable at physiological levels. In addition, an electrocardiogram (ECG) was used to identify left ventricular end-diastolic volume (LVEDV) and left ventricular end-systolic volume (LVESV) triggers. B-mode data were acquired in the parasternal long axis view (PSLAX). For reconstructed 3D-echo images, the transducer was positioned perpendicular to the long-axis view (short-axis view) and the left ventricle was scanned by consecutive images (0.064 mm intervals) of the short axis using a 3D-motor (visual sonics). 3D images were reconstructed based on the drawing of approximately 10-12 volumes of interest from apex to base (see Supplemental Figure 2). LVEDV and LVESV were used to calculate left ventricular ejection fraction (LVEF).

Post measurement speckle tracking based analysis were performed to determine myocardial deformation parameters (VevoStrain, VisualSonics). Echocardiographic images acquired from the PSLAX were used to measure peak velocity (cm/s), strain (%) and strain rate (1/s) in the longitudinal and radial axis.

Three consecutive cardiac cycles were selected for analysis and semi-automated tracing of the endocardial and epicardial border was performed resulting in 48 sampling points. The myocardium was automatically divided in 6 segments for regional speckle-tracking analysis; basal-anterior (BA), mid-anterior (MA), apical-anterior (AA), apical-posterior (AP), mid-posterior (MP) and basal-posterior (BP). Global measurements were defined as the average of all 6 segments, whereas the infarct and remote area were defined as the average of MA, AA, AP and MP, BP respectively.

**Histology**

At day 28 after intramyocardial injection with either CPC or vehicle, mice were terminated by exsanguination under general anesthesia and their hearts were excised. The hearts were dehydrated and fixed in a 15% sucrose 0.4% PFA solution after which they were embedded in O.C.T. compound (Tissue Tek) and stored at -80 °C. Serial transverse cryosections of 7 μm were cut, base to apex, for histological and immunohistological stainings, which were performed by a blindend investigator.

Sections were stained in haematoxylin solution (Boom) and eosin solution (BDH) (H&E), followed by dehydration and embedding in entellan. H&E stainings were used for quantification of the infarct size. Next, picrosirius red staining was performed to analyze the collagen composition and density in the infarcted area. Stained sections were imaged with light microscopy to define the infarct area and with polarized light microscopy to analyze collagen fibers. The infarct area was assessed visually as the area with disrupted myocardium. Collagen density is expressed as mean grey value per mm^2^ infarct area. Analyses were performed with CellSense (Olympus).

Engraftment of human CPC and the effect on matrix composition was assessed by immunostaining and the visual aspect of the cardiomyocyte pattern as background layer. Cryosections were incubated for 1 hour at room temperature with primary antibodies against: human Lamin A/C antibody (1:100, VP-L550, Vector), Troponin-I (1:50, sc-15368, Santa Cruz), vimentin (1:100, sc-7557, santa cruz), α-SMA (1:100, F3777, Sigma), collagen type 1 (1:50, 600-4010103S, Rockland), collagen type 3 (1:50, ab-7778, Abcam), CD31 (1:50, 550274, eBioscience), CD45 (1:50, BD Pharmingen). Subsequently, sections were incubated o/n at 4°C using the appropriate Alexa 488 and Alexa 555 antibody (Invitrogen). Nuclei were stained with Hoechst (33342). For clear CD31 staining, an amplification step with biotin/streptavidin (DAKO) was included.
