# Supplementary figures and images for: Targeting chronic cardiac remodeling with cardiac progenitor cells in a murine model of ischemia/reperfusion injury
Source: PLoS One. 2017 Mar 20;12(3):e0173657. doi: 10.1371/journal.pone.0173657 (PMC5358772; doi:10.1371/journal.pone.0173657)

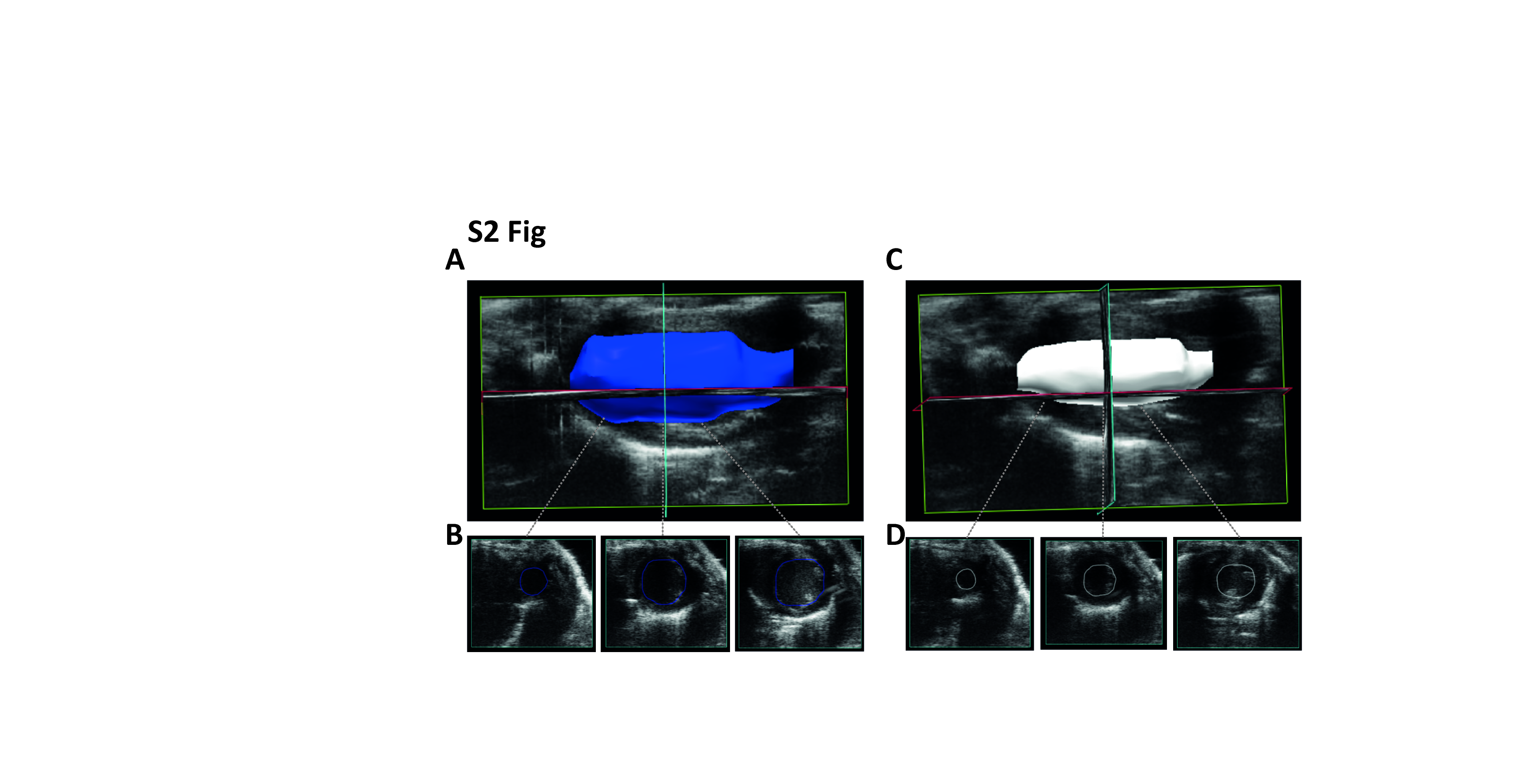

Supplement: S2 Fig — (A) Representative images of the contours of the LVEDV and (B) LVESV in a reconstructed 3D echocardiographic image. Bottom panels show examples of the contours drawn in the short axis images used to reconstruct the left ventricle. (TIFF) [file pone.0173657.s003.tiff]
