# Supplementary material for: Targeting chronic cardiac remodeling with cardiac progenitor cells in a murine model of ischemia/reperfusion injury
Source: PLoS One. 2017 Mar 20;12(3):e0173657. doi: 10.1371/journal.pone.0173657 (PMC5358772; doi:10.1371/journal.pone.0173657)
Supplement: S1 Table — All 6 myocardial segments were used for quantification of velocity, strain and SR. p-values in the baseline column denote differences between baseline and 28 days I/R. In addition, p-values in the pre-treatment and post-treatment column show differences between the CPC and vehicle group. (DOCX) [file pone.0173657.s005.docx]

| Regional deformation | baseline | | pre-treatment | | | | post-treatment (delta) | | |
| --- | --- | --- | --- | --- | --- | --- | --- | --- | --- |
|  | overall |  | overall (n=29) | CPC (n=13) | Vehicle (n=16) |  | CPC (n=13) | Vehicle (n= 16) |  |
| Radial |  |  |  |  |  |  |  |  |  |
| velocity (cm/s) | 1.4±0.08 | **0.0013** | 1.10±0.05 | 1.0±0.07 | 1.2±0.07 | **0.0471** | 0.1±0.04 | -0.1±0.06 | **0.0042** |
| strain (%) | 35.3±3.3 | **0.0017** | 23.8±1.7 | 21.7±2.4 | 25.5±2.4 | 0.2813 | 4.4±2.2 | 0.02±2.4 | 0.2065 |
| SR (1/s) | 9.1±0.6 | **0.0001** | 6.0±0.4 | 5.2±0.5 | 6.6±0.6 | 0.0905 | 0.8±0.5 | -0.7±0.5 | **0.0394** |
| Longitudinal |  |  |  |  |  |  |  |  |  |
| velocity (cm/s) | 0.6±0.05 | 0.2444 | 0.5±0.03 | 0.6±0.03 | 0.6±0.05 | 0.7793 | -0.1±0.06 | -.02±0.05 | 0.3123 |
| strain (%) | -16.4±1.8 | 0.1017 | -13.8±0.7 | -13.6±0.6 | -13.9±1.2 | 0.7999 | 1.2±0.9 | 1.6±1.6 | 0.8027 |
| SR (1/s) | -5.7±0.4 | **0.0481** | -4.6±0.3 | -4.3±0.3 | -4.9±0.5 | 0.2937 | 0.3±0.3 | 0.8 ±0.5 | 0.3949 |

**S1 Table. Global myocardial deformation.**
